# An Online Survey of Australian Medical Students’ Perspectives on Spiritual History Taking and Spiritual Care

**DOI:** 10.1007/s10943-023-01897-2

**Published:** 2023-09-19

**Authors:** John Wenham, Megan Best, David W. Kissane

**Affiliations:** 1 Broken Hill Department of Rural Health, Sydney Medical School, Corrindah Court, PO Box 457, Broken Hill, NSW 2880 Australia; 2 Institute for Ethics and Society, The University of Notre Dame Australia, 104 Broadway, PO Box 944, Broadway, NSW 2007 Australia; 3 Palliative Medicine Research, The University of Notre Dame Australia, Broadway, NSW 2007 Australia; 4 The Cunningham Centre for Palliative Care Research, St Vincent’s Hospital, Sydney, NSW Australia

**Keywords:** Spiritual care, Spiritual history taking, Communication skills, Medical students

## Abstract

It is reported that little spiritual care communication skills training occurs in Australian medical schools. This survey explored the experience of final year students in this domain in order to inform the construction of a new curriculum. Medical students in their final year at four Australian medical schools were invited to participate in an online survey, which included questions about demographic details, exposure to spiritual history taking, perceived learning needs, and the Functional Assessment of Chronic Illness Therapy-Spiritual Well-being 12 item Non-Illness score. Two-hundred and sixty students from a cohort of 766 responded (34%). One in nine students had witnessed spiritual history taking, and one in ten students had been given the opportunity to do so. Barriers and enablers were identified. Two-thirds of the students reported no recollection of any training in spiritual care. When it did occur, it was limited in scope and structure. Final year medical students recognise that spiritual care deserves a place in the modern, broad-based medical school curriculum. This supports the argument for inclusion of spiritual care training as part of all medical student curricula in Australia.

**Supplementary Information:**

The online version contains supplementary material available at 10.1007/s10943-023-01897-2.

## Introduction

Holistic medicine should be provided to patients in whatever context they present, from preventive medicine in General Practice, through to emergency departments, psychiatry clinics, and palliative care (Chidarikire, 2012; Thomas et al., 2018; Vissers et al., 2013). Holistic medicine will ideally address all dimensions of human experience (Thomas et al., 2018). However, research has shown discordance between patients’ desires in the domain of spiritual care and what is provided by their doctors.

“Spirituality has been defined as the dynamic dimension of human life that relates to the way persons (individual and community) experience, express and/or seek meaning, purpose and transcendence, and the way they connect to the moment, to self, to others, to nature, to the significant and/or the sacred” (Nolan et al., 2011; Puchalski et al., 2014a). Best et al., (2016a, 2016b), in a systematic literature review of 54 studies comprising 12,327 patients, demonstrated that a median of 70% of patients declared an interest in discussing spirituality with their physicians. However, only a median of 15% of consultations with their doctors, from a broad range of GP, acute, and life-limiting settings actually included it.

In addition, there is clear evidence that spiritual well-being improves patient health outcomes (Burlacu et al., 2019; Park et al., 2015). If spiritual care is high on the patient agenda, in whatever clinical context (standard GP consultation through to a palliative care setting), and remains an unmet need, their care will have been suboptimal. It is more likely to be addressed if the doctor has received appropriate training (Best et al., 2016a). The need for spiritual care exists across cultures and religions. In a chapter on spiritual care in the *Handbook to Healthcare in the Arab World* (2021), it is stated that there is currently a dearth of published research on this topic from Arab countries. However, the authors propose several recommendations to improve the assessment and provision of spiritual care within Arab health-care systems. Examples include increasing the number of staff, offering training to staff on assessing and providing spiritual care, and adopting new polices to ensure spiritual care is provided to Arab patients (Abu-El-Noor & Abu-El-Noor, 2021).

Overall, from the broad range of excellent work in the USA*,* South America, and to a lesser extent, in Europe, it seems clear that where spirituality is included in the medical education curriculum, barriers to spiritual care are reduced, skills in history taking and exploring of holistic care are enabled, and medical trainees are better equipped to understand their own needs for spiritual self-care. Authors of these studies suggest that this content needs to be included in the standard medical school curriculum (Puchalski et al., 2014b; Schonfeld et al., 2016). In 2010 a national survey in the USA reported that 90% of medical schools include courses or content on spirituality and health (Koenig et al., 2010). Anecdotally, it seems to occur infrequently in Australian medical curricula; it may include a spiritual care lecture or a palliative care elective in which a limited number of students from each cohort participate (Bennett et al., 2014).

Spiritual care training also has benefits for clinicians. Chow et al. (2021) carried out a global scoping review of spirituality and religion in American hospital residents in order to examine the impact of these factors on clinical care and on the residents’ own psychological well-being. Residents held positive attitudes about the role of spirituality and religion, and the impact on patient care—better therapeutic relationship, treatment adherence and coping with illness although they often lacked the knowledge and skills to address them. Higher spiritual well-being of residents was associated with greater sense of work accomplishment, greater overall self-rated health, decreased burnout, and depressive symptoms.

The timing of spiritual care has also been examined. Research shows that spiritual discussion is often neglected by doctors until patients are approaching the end of life. However, it might be avoided even then because of insufficient physician knowledge and training, fear that the physician would not be able to manage any issues raised and personal discomfort of the physician with the topic (Best et al., 2016a, 2016b). Luckhaupt et al. (2005) found that 77% of US doctors in primary care residency agreed they should ask about patient beliefs if the patient were near death, but only 36% felt they should ask during a standard office visit. Rombola (2019) surveyed a cohort of 147 Australian doctors, 53% of whom were GPs. Forty-seven per cent included spiritual assessment in consultations at least occasionally, but only 21% felt confident in doing so.

Spiritual care of patients is important if physicians wish to practice whole person care. Humans have physical, emotional, social, and spiritual needs. In order to flourish, each aspect needs to be working well. Borneman et al. (2010) argued that, “Addressing spiritual needs and concerns in clinical settings is critical in enhancing quality of life”. This gap needs to be addressed in order to care for patients in a way that improves outcomes and reflects their wishes. It can therefore be argued that spiritual care should be included in the core teaching of our medical schools. If this foundational work is done well, it will be possible to develop previously learnt communication skills further in residency programs.

### Spiritual History Taking

Current basic communication skills training tends to focus on breaking bad news and facilitating the extraction of information from patients in the GP setting. Furthermore, there is evidence that final year basic communication skills are unlearnt quickly and junior doctors tend to copy poor communication practices that they observe in senior doctors (Cruess et al., 2008). Spiritual care requires advanced communication skills (Batstone et al., 2020), and therefore these skills will need to be taught so that medical students are equipped to be able to assess the spiritual needs of patients effectively.

A formal spiritual history involves asking specific questions during a medical interview to determine the importance of spirituality in a patient’s life, whether spiritual factors may play a role in the patient's illness or recovery and whether these factors affect the medical treatment plan. In clinical practice, the spiritual history is usually performed early in the relationship with a patient, and clinicians may reassess the patient regularly to check whether a spiritual problem develops. Should the clinician identify a spiritual problem at any stage, they can then refer to a chaplain or other spiritual care practitioner, who will perform a full spiritual assessment (Drummond & Carey, 2019). This practice should be distinguished from triage conducted by nurses or social workers in countries such as the USA, which often includes screening for spiritual distress.

In order to train Australian medical students to administer spiritual care, a curriculum needs to be developed including, in particular, the art of spiritual history taking. Spiritual history taking is a key skill for medical students to develop, and for doctors to cultivate, in order to practice holistic and culturally sensitive medicine effectively and competently (Puchalski et al., 2009; Rombola, 2019).

When first learning to take a spiritual history, a tool can be helpful for students. There are many possible formats for conducting a formal spiritual history, such as the HOPE and FICA models. HOPE covers four concepts:- (i) sources of Hope; (ii) the role of Organised religion for the patient; (iii) Personal spirituality and practices; and (iv) Effects on medical care and end-of-life decisions (Anandarajah & Hight, 2001). The FICA framework comprises:- (i) Faith or belief; (ii) Importance and influence of spirituality; (iii) Community; and (iv) Address spiritual needs (Borneman et al., 2010).

A spiritual care curriculum is anticipated to require a half-day workshop in addition to standard communication skills training. This is because in their paper, Best et al., (2016a, 2016b) described spiritual history taking as, “a delicate, skilled, tailored process whereby physicians create a space in which patients feel safe enough to discuss intimate topics”. Interviews with physicians experienced in spiritual care revealed that, in order to develop expertise in spiritual care, they needed to develop their own spirituality and learn to appreciate the importance of spirituality for patients as first steps. Studies have found that the doctor most likely to address religion and spirituality in a medical consultation has strong intrinsic and extrinsic spirituality and religiosity (Curlin et al., 2005, 2006).

Before developing a spiritual care curriculum, it is helpful to understand what is currently being taught in medical schools, and what is currently understood by medical students with regard to spiritual care. It is also helpful from the perspective of pedagogy to identify student characteristics that may enhance spiritual care capability. This survey was therefore conducted to inform the design of future medical curricula within Australian universities.

## Aims

This study sought to:

1. Explore final year medical students’ understanding of spiritual care;

2. Assess current teaching of spiritual care;

3. Evaluate students’ spiritual care learning needs;

4. Explore barriers and enablers of spiritual care;

5. Analyse whether there are associations between the students’ level of spirituality and their desire to engage with spiritual care.

## Methods

The Deans of Medicine at the Universities of Adelaide, Notre Dame, Sydney and Wollongong were contacted in June 2021 to seek their support for this project. These universities have schools located across three states (Western Australia, South Australia and New South Wales) and provide a mix of both established and recently developed institutions and city/regional/rural student training locations. The Deans all wrote letters of support which were submitted with the ethics application.

An online questionnaire-based survey was designed to elicit medical students’ understanding of and learning needs in spiritual care, their level of confidence in taking spiritual histories, their current practice, and barriers and enablers of spiritual care. Questionnaire development was informed by the literature (Peterman et al., 2002; Rombola, 2019) and the personal experience of the researchers (Wenham et al., 2021).

Demographic data collected included spiritual/religious worldview and level of participation in religious practices. Spiritual well-being was assessed using the Functional Assessment of Chronic Illness Therapy—Spiritual Well-Being 12 Item Scale Non-Illness score (FACIT-Sp12 NI), (Bredle et al., 2011). The FACIT-Sp12 NI is a validated measure to ascertain religiosity and global spirituality. Spirituality is measured by three sub-scales (meaning, peace, and faith).

Students were also asked about their views regarding taking a spiritual history, the importance of spiritual care training, and how it should be conducted in the medical curriculum (see Online Appendix). The questionnaire was piloted with ten medical students at the lead author’s rural teaching site to ensure that it was comprehensible and feasible. Responses were given either by multiple choice options or Likert scales, depending on the question being asked. These responses were collated electronically via REDCap.

Ethics approval was obtained from the University of Notre Dame Human Research Ethics Committee [Reference Number: 2021-116S] and circulated to the respective ethics departments of the other three participating medical schools for their records. The Deans then circulated the questionnaire via e-mail to their final year cohorts in late 2021. This was repeated in early 2022 to achieve a higher number of participants. Power analysis suggested that with 20 items/sub-scales to assess, 10 participants required per item/sub-scale, and a total of 200 complete surveys would be required. The survey was closed on 17 May 2022.

Seven-hundred and sixty-six medical students were in their final year of study across the four campuses. They were all sent an electronic link to the questionnaire, either directly to their e-mail or a notification posted on their campus electronic learning sites (e.g. Canvas). Consent to participate was given at the commencement of the electronic survey.

Descriptive statistics were analysed within the REDCap platform. STATA v15 was used for correlational analyses. Comparisons were made using a Pearson’s correlation between each student’s FACIT-Sp12 NI scores and their willingness to discuss spiritual matters, whether spiritual care is important and whether they should receive training.

## Results

A total of 260 questionnaires had complete data (total response rate 34%). Women made up almost 60% of the participants. Table 1 shows the demographics of the group.

**Table 1:** Final year medical school student demographics

Characteristic	Number (260)	%
Women	151	58
Age (mean) in years	26.9	
*Medical School*		
Adelaide	44	17
Notre Dame	33	13
Sydney	139	53
Wollongong	44	17
*Ethnicity*		
Oceanian	47	18.1
European	120	46.2
African & Middle Eastern	14	5.4
Asian	68	26.2
Other	10	3.8
*Spiritual Worldview*		
Humanism	83	31.9
Naturalism	58	22.2
Pantheism	18	7
Polytheism	2	0.8
Theism	73	28
Post-modernism	26	10.1
*Religious Worldview*		
Atheist	64	24.6
Agnostic	112	43.1
Polytheistic	6	2.7
Monotheistic	77	29.6
*Religious Affiliation*		
Buddhist	12	4.6
Christian	99	38.1
Hindu	10	3.8
Jewish	3	1.2
Muslim	9	3.5
Other	10	3.8
None	117	45
*Participation in religious practice*		
Open to spiritual matters	126	48.5
Belong to religious community	54	20.8
Attend place of worship at least monthly	38	14.6
Active daily participant in spiritual activity	46	17.7
None	106	40.8

Response rates varied across the four universities with 44/137 Adelaide students (32%), 33/249 Notre Dame students (13%), 139/300 Sydney students (46%), and 44/80 Wollongong students (55%) participating. The higher rates from Sydney and Wollongong were achieved by the lead author having the opportunity to speak about the purpose of the research and inviting student participation directly.

This cohort reported a broad range of spiritual and religious worldviews. Humanism (31.9%) was more prevalent than both theism (28%) and naturalism (22.2%) within spiritual worldview. Agnostics (43.1%) held the dominant religious worldview, followed by monotheists (29.6%) and atheists (24.6%). The predominant religious affiliation was none (45%) with Christianity second (38.1%).

One in nine students recalled witnessing a colleague takes a spiritual history (11%), and one in ten students had been given the opportunity to do so (10%). Two-thirds of the students did not recall receiving any training in spiritual care or spiritual history taking (66%) (Table 2). Those who had been trained formally remembered the use of the modalities of small group or case-based discussions and being taught the components of a spiritual history. Thirteen per cent of students had training in their first year at medical school, but only 10% had training in clinical years (between third and fifth years).

**Table 2:** Student experience of spiritual care

Nature of experience	Range of responses	Number (260)	%
Have you ever seen a clinician take a spiritual history from a patient?	Yes	28	11
	No	230	89
During your training, have you ever been given the opportunity to take a spiritual history?	Yes	26	10
	No	232	90
Have you ever had any training in spiritual history taking or spiritual care?	Previous course	14	5.4
	First year	33	12.7
	Second year	39	15
	Third year	12	4.6
	Fourth year	11	4.2
	Fifth year	2	0.8
	Never	178	68.5
What did the training involve? (Select as many as apply)	Spiritual history taking	37	41.6
	Chaplain shadowing	4	4.5
	Teaching OSCE	2	2.2
	Case-based discussions	22	24.7
	Simulated patients	3	3.4
	Small group discussions	32	36
	Self-care	16	18
	Comparative religions	8	9
	Self-reflective journaling	11	12.4
	Other	17	19

The majority of final year medical students were ambivalent about discussing spiritual matters with patients; 26% were inclined to discuss them and 11% were against the idea. Over half of the students stated that insufficient knowledge, insufficient training, and lack of time were barriers to discussion (Table 3). Fear of crossing professional boundaries, disapproval of colleagues, and being unable to manage the issues raised by patients were significant concerns. Free text responses also included the following:

- “It doesn’t seem appropriate”.
- “I’m not sure it’s a doctor’s role”.
- “The patient may get uncomfortable with the topic”.
- “I’m unsure of the legality around it [taking a spiritual history]”.
- “Only ask if necessary – if it relates to their presentation or treatment”.
- “I have a total personal disinterest in the topic”.

**Table 3:** Student attitudes about future participation in spiritual care

Attitude	Responses	Number	%
How do you feel about discussing spiritual matters with patients?	Strongly inclined to	9	3.5
	Inclined to	59	22.8
	Neutral	162	62.5
	Against	22	8.5
	Strongly against	7	2.7
What would prevent you from exploring spiritual matters with a patient?	Insufficient knowledge	135	53
	Insufficient training	148	58
	Fear of being unable to manage issues raised	109	43
	Personal discomfort with the task	54	21
	Uncertainty of your own spirituality	21	8
	Fear of crossing doctor/patient boundary	111	43
	Time limitations	154	60
	Fear of disapproval of colleagues	62	24
	I don’t believe it is a doctor’s role	40	16
	Other	13	5
What would increase your likelihood of exploring spiritual matters with a patient?	Patient raises the issue—person-centred	227	88
	End of life scenario	204	79
	I’m a spiritually sensitive person	45	17
	Positive previous experience	88	34
	Communication skills training	85	33
	Previous spiritual care training	109	42
	Aware of its importance in the patient’s life	176	68
	Culturally important to the patient	182	70
	Other	11	4
Spiritual care is important within the medical consultation	Strongly disagree	5	1.9
	Disagree	27	10.4
	Neutral	97	37.5
	Agree	101	39
	Strongly agree	29	11.2
Medical students should receive training in this field as part of their core curriculum	Strongly disagree	8	3.1
	Disagree	19	7.3
	Neutral	88	34
	Agree	114	44
	Strongly agree	30	11.6
At what stage in training would you think it best to include spiritual care?	Pre-clinical	37	15
	Early clinical	90	35
	Late clinical	97	38
	During a junior doctor training program	32	12
When is the best time for a doctor to raise spiritual matters with a patient?	First time seen	35	13
	Subsequent visits	77	30
	Health crisis	25	10
	End of life	74	29
	Hospital admission	5	2
	Never	7	3
	Other	36	14

Strong enablers of engagement with spiritual care included patients facing the end of life, patients raising the issue, and understanding the importance of this part of an individual patient’s life. Students also thought that good communication skills and spiritual care training would help them get involved. Fifty per cent of students agreed that spiritual care was important in the medical consultation; only 12% disagreed. More than half of this cohort agreed that spiritual care should be taught as part of the core medical school curriculum; only 10% disagreed. Overall, there was a trend towards identifying the latter part of the clinical years as the best timing for the training.

When asked to choose between training components used in medical school curricula internationally, strong support was given to spiritual history taking skills, small group or case-based discussions, and role playing with simulated patients (Table 4).

**Table 4:** Student-preferred curriculum components

Suggested component	Priority	Total (260)	%
Spiritual history taking tutorial	1	177	70
Case-based discussions	2	148	58
Small group discussions	3	132	52
Simulated patient role plays	4	122	48
Chaplain shadowing	5	87	34
Comparative religions	6	80	31
Teaching OSCE	7	61	24
Self-care	8	53	21
Self-reflective journaling	9	25	10

Wenham et al., 2021

When a comparison was made between the FACIT-Sp12 NI scores for female and male students, the correlation for each question was greater in women. Students who have higher spiritual well-being (higher FACIT-Sp12 NI scores) were significantly more open to the discussion of spiritual matters within the medical consultation (*p* < 0.001). They were also significantly more likely to believe that spiritual care is important (*p* < 0.001). These correlations reached statistical significance (Table 5).

**Table 5:** Correlation between FACIT-Sp12 NI scores and views on spiritual care

Attitude	Correlation coefficients with FACIT-Sp12 NI
How do you feel about discussing spiritual matters with a patient?	0.275^**^
Spiritual care is important within the medical consultation	0.240^**^
Medical students should receive training in this field as part of their curriculum	0.170^*^

^*^ *p* < 0.05, ^**^ *p* < 0.001

## Discussion

This study reported the views of final year medical students from four different Australian universities regarding their current understanding of spiritual care, and how their learning needs might be addressed in a new curriculum.

### Current Practice of Spiritual Care

The students reported limited exposure to clinicians’ modelling of spiritual history taking, and only 10% recalled having had the opportunity to do this themselves. Almost 70% of students had not received training in any aspect of spiritual care by the time they reached their final year of Medical School. If they had, it was limited to spiritual history taking and case-based discussions with a small number reporting exposure to chaplain shadowing (4.5%) or simulated patients (3.4%). Overall, given the importance of person-centred care, this demonstrates a deficit in current medical school curricula within Australian universities.

### Understanding of Spiritual Care

Despite their limited clinical experience in this domain, 50% students agreed that spiritual care was an important part of the medical consultation, with only 12% disagreeing. Fifty-five per cent believe that medical students should receive training in this field as part of their core curriculum, whilst only 10% disagreed. This demonstrates that final year students affirm the importance of the subject matter and understand the need to be equipped to care for patients’ spiritual needs.

### Learning Needs

When asked to select from nine possible components of medical education on spiritual care, more than 50% of the students chose the top three options. These were spiritual history taking tutorials, case-based discussions, and small group discussions. Any future curriculum design should address the expressed desire to be able to take a competent spiritual history, and case-based and small group discussion platforms would be helpful in achieving this.

### Barriers to Spiritual Care

Examination of the reported barriers to exploring spiritual matters with a patient showed insufficient knowledge, lack of training, and the limitations of time as the most significant. The first two issues can be addressed with well-timed medical education. The free text responses to this question gave further insights with students expressing concerns about appropriateness to patient presentation or treatment, legality, relevance to their role as a doctor, and the possibility of patient discomfort with the topic. We suggest that a curriculum that informs medical students of the scope of their role, and provides evidence of patients’ wishes and experiences in the domain of spiritual care, would give greater confidence to them to engage. Stating, “I have no personal interest in the topic,” is an attitudinal issue which would be a barrier to care in a variety of situations where the doctor and patient values or priorities did not align.

Even though time is frequently mentioned as a barrier to doctors providing spiritual care, research has shown that, even when more time is available, doctors still do not ask about patient spirituality. Therefore it is unlikely to be a true barrier and may be resolved with education that shows that enquiring about spirituality does not need to be time-consuming (Best et al., 2016a; Olson et al., 2006).

### Enablers of Spiritual Care

Students reported enablers of exploring spiritual matters with a patient as:- when these are raised by the patient; when it is known to be important to the patient; when culturally it would be appropriate; or when the end of life is approaching. Spiritual care training would improve confidence as well as encouraging spiritually sensitive practitioners (Best et al., 2016a). One free text comment from a participant simply stated, “If it is deemed an important part of medical practice and becomes part of training as a doctor, I would”. Responding to cues from a patient that suggest they are comfortable with spiritual matters was also deemed appropriate. This method has been found to be successful for initiating spiritual conversations by experienced practitioners (Best et al., 2016a). We found that students reported wanting to be able to learn to read the environment and that they believe training will assist them in this.

### Timing of Training

With regard to deciding the best time during medical school to incorporate spiritual care training, students favoured the late clinical years over early clinical and pre-clinical. This sits well with the authors, given the complexity of the task being considered. Addressing spiritual care after students have gained some clinical experience would also make it more likely that students would realise the relevance of it in the clinical context.

### Respondents’ Spirituality

Our student sample was broadly representative of the wider population’s religious views, apart from 5% fewer Christians and a reciprocal increase in agnostics. The 2021 Australian census data demonstrated the religious affiliation of the population as:- none 38.9%, Christian 43.9%, Buddhist 2.4%, Muslim 3.2%, and Hindu 2.7%.

Students’ personal spiritual well-being (calculated from FACIT Sp-12 NI scores) correlated with openness to and perception of the importance of spiritual assessment; females had a stronger correlation to this than males. In their study, Galek et al. (2008) found that females experienced five of the seven spiritual constructs they measured significantly more often than men. Higher spiritual well-being scores did not correlate with strong support for its inclusion in the curriculum. Thus, we might hypothesise that the broad-based support from 55% of students surveyed is not dependent on any particular spiritual or religious affiliation. This lends greater strength to the argument that inclusion of this training in Medical School curricula would be a benefit to the majority of future doctors and consequently their patients.

## Limitations

Although our response rate was only 34%, we believe that in the context of the busy modern medical student’s email inbox which is besieged with surveys after almost every learning experience, this is a good response rate for a voluntary survey. Furthermore, our sample was more than sufficient to power our study.

We believe that our responses are representative of final year students across Australia. Our sample includes two capital cities (Sydney and Adelaide), a regional city (Wollongong) and rural settings from Notre Dame and Adelaide medical schools, with a similar religious profile to the population. The broad representation of multicultural students with diverse world views also increases the legitimacy of the findings.

It is likely that this survey would have been subject to respondents’ self-selection bias. This may have led to an upward distortion of the actual proportion of medical students supportive of the inclusion of spiritual care in their training programs. However, our student sample closely matches the 2021 census data for religious affiliation.

Whilst it is true that the FACIT-Sp12 NI has only been validated in the clinical setting, it has previously been used to assess clinicians’ level of spirituality. It therefore seemed reasonable to the authors to use it to assess final year medical students in this domain (Cella, 2010). It is also true to say that there is overlap, contamination, and possibly even inseparability, between spirituality/religious belief and mental health status. Scales to measure extrinsic distress are subject to the same challenge. In part we have to accept this as holistic medicine recognises the inter-relationship between body, mind, and spirit. However the FACIT-Sp12 NI is a validated tool for such an assessment. One way to mitigate this in future studies would be to employ a second tool such as Bryson’s (2015) Spiritual Assessment System.

## Conclusion

It is reassuring to find that final year medical students recognise, firstly, the absence of spiritual care training from their curricula and, secondly, perceive its benefits to the greater development of person-centred care. There is work to be done in designing such a curriculum that will meet their learning needs.

More than half of these students believe that spiritual care is important within the medical consultation. The students recognise that spiritual care deserves a place in the modern, broad-based medical school curriculum; in fact, only 10.3% students are opposed to its inclusion. There was a trend towards spiritual care training being delivered in the later stages of clinical training. This aligns with the view that this involves higher-level communication skills.

We believe there is a strong argument to include spiritual care training as part of all medical student curricula in Australia; this should no longer just be for those who participate in a palliative care elective placement. We have commenced work on a broader proposal which we plan to publish in the near future.

### Supplementary Information

Below is the link to the electronic supplementary material.

Supplementary file1 (DOCX 34 KB)
