# Supplementary material for: An Online Survey of Australian Medical Students’ Perspectives on Spiritual History Taking and Spiritual Care
Source: J Relig Health. 2023 Sep 19;63(1):257–73. doi: 10.1007/s10943-023-01897-2 (PMC10861599; doi:10.1007/s10943-023-01897-2)
Supplement: Supplementary file 1 — Supplementary file1 (DOCX 34 KB) [file 10943_2023_1897_MOESM1_ESM.docx]

**APPENDIX**

**Final year medical student perspectives on spiritual history taking and spiritual care**

A definition of **spirituality** was agreed at a consensus conference in 2009: “Spirituality is the aspect of humanity that refers to the way individuals seek and express meaning and purpose and the way they experience their connectedness to the moment, to self, to others, to nature, and to the significant or sacred.’’(1)

**Religion** is belief in a [God](https://www.collinsdictionary.com/dictionary/english/god) or gods and the activities that are [connected](https://www.collinsdictionary.com/dictionary/english/connect) with this belief, such as [praying](https://www.collinsdictionary.com/dictionary/english/pray) or worshipping in a building such as a church or [temple](https://www.collinsdictionary.com/dictionary/english/temple). [Collins English Dictionary]

**Questions**

1. What is your gender?
2. What is your age in years?
3. Where are you currently studying medicine?
   - - Adelaide Medical School
     - Notre Dame Medical School
     - Sydney Medical School
     - Wollongong University
4. How would you best describe your spiritual worldview? Choose one. See Appendix 1 if you require descriptors. (2)
   - Humanism
   - Naturalism
   - Pantheism
   - Polytheism
   - Theism
   - Post-modernism
5. Please describe your own religious world view. Choose one:

- Atheist – there is no god
- Agnostic - unsure
- Polytheistic – believe in many gods
- Monotheistic – believe in one God

1. What is your religious affiliation?

- Buddhist
- Christian
- Hindu
- Jew
- Muslim
- None (go to question 8)
- Other, please specify

1. What level of participation do you have in religious practices?

- Open to spiritual matters
- Belong to a religious community
- Attend place of worship at least monthly
- Active daily participant in spiritual activities

1. What is your ethnic origin?

- Oceanian
- Northwest European
- Southern and Eastern European
- North African and Middle Eastern
- Southeast Asian
- Northeast Asian
- Southern and Central Asian
- Peoples of the Americas
- Sub-Saharan African
- Prefer not to say

1. Have you ever seen a clinician take a spiritual history from a patient?

- YES
- NO

10. During your training, have you ever been given the opportunity to take a spiritual history?

- 1. YES
  2. NO

11. How do you feel about discussing spiritual matters with patients?

- Strongly inclined to
- Inclined to
- Neutral
- Against
- Strongly against

12. Have you ever had any training in spiritual history taking or spiritual care?

Tick as many as apply.

- Previous course – please state
- First year
- Second year
- Third year
- Fourth year
- Fifth year
- Never

13. If yes, what did it involve? Tick as many as apply.

- Spiritual history taking
- Chaplain shadowing
- Teaching OSCE
- Case based discussions
- Simulated patients
- Small group discussions
- Self-care
- Comparative religions
- Self-reflective journaling
- Other (please specify)

14. What would prevent you from exploring spiritual matters with a patient?

Select as many as apply:

- Insufficient knowledge
- Insufficient training
- Fear of being unable to manage issues raised
- Personal discomfort with the task
- Uncertainty of your own spirituality
- Fear of crossing doctor/patient boundary
- Time limitations
- Fear of disapproval of colleagues
- I don’t believe it is a doctor’s role
- Other – please specify

15. What would increase your likelihood of exploring spiritual matters with a patient?

- Patient raises the issue – person-centred
- End of life scenario
- I’m a spiritually sensitive person
- Positive previous experience
- Communication skills training
- Previous spiritual care training
- Aware of its importance in the patient’s life
- Culturally important to the patient
- Other – please specify

16. Spiritual care is important within the medical consultation:

- Strongly disagree
- Disagree
- Neutral
- Agree
- Strongly agree

17. Medical students should receive training in this field as part of their core curriculum?

- Strongly disagree
- Disagree
- Neutral
- Agree
- Strongly agree

18. At what stage in training would you think it **best** to include spiritual care?

- Pre-clinical
- Early clinical
- Late clinical
- After medical school during a junior doctor training program

19. When is the best time for a doctor to raise spiritual matters with a patient?

- First time seen
- Subsequent visits
- Health crisis
- End of life
- Hospital admission
- Never – please specify
- Other – please specify

20. Which of the following components do you think would be effective in medical education on spiritual care?

|  | Yes | Maybe | No | Comments |
| --- | --- | --- | --- | --- |
| Spiritual history taking tutorial |  |  |  |  |
| Chaplain shadowing |  |  |  |  |
| Teaching OSCE |  |  |  |  |
| Case based discussions |  |  |  |  |
| Simulated patient role plays |  |  |  |  |
| Small group discussions |  |  |  |  |
| Self-care |  |  |  |  |
| Comparative religions |  |  |  |  |
| Self-reflective journaling |  |  |  |  |

21. Here we ask about aspects of your quality of life and wellbeing

*Please circle or mark one number per line to indicate your response as it applies to the* ***past 7 days.***

|  | Not at all | A little bit | Somewhat | Quite a bit | Very much |
| --- | --- | --- | --- | --- | --- |
| 1. I feel peaceful | 0 | 1 | 2 | 3 | 4 |
| 2. I have a reason for living | 0 | 1 | 2 | 3 | 4 |
| 3. My life has been productive | 0 | 1 | 2 | 3 | 4 |
| 4. I have trouble feeling peace of mind | 0 | 1 | 2 | 3 | 4 |
| 5. I feel a sense of  purpose in my life | 0 | 1 | 2 | 3 | 4 |
| 6. I am able to reach down deep into myself for comfort | 0 | 1 | 2 | 3 | 4 |
| 7. I feel a sense of harmony within myself | 0 | 1 | 2 | 3 | 4 |
| 8. My life lacks meaning and purpose | 0 | 1 | 2 | 3 | 4 |
| 9. I find comfort in my faith or spiritual beliefs | 0 | 1 | 2 | 3 | 4 |
| 10. I find strength in my faith or spiritual beliefs | 0 | 1 | 2 | 3 | 4 |
| 11. Difficult times have strengthened my faith or spiritual beliefs | 0 | 1 | 2 | 3 | 4 |
| 12.Even in difficult times I know that things will be okay | 0 | 1 | 2 | 3 | 4 |

References

1. Puchalski, C. M., Ferrell, B., Virani, R., Otis-Green, S., Baird, P., Bull, J., Chochinov, H., Han-dzo, G., Nelson-Becker, H., Prince-Paul, M., Pugliese, K., & Sulmasy, D. (2009). Improving the quality of spiritual care as a dimension of palliative care: The report of the consensus conference. *Journal of Palliative Medicine, 12*(10), 885-904. https://doi.org/10.1089/jpm.2009.0142
2. Six Worldviews accessed 26/07/21 from:

<https://rw360values.org/wp-content/uploads/2019/10/Six-Worldviews.pdf>
